# Tamoxifen decreases ovarian toxicity without compromising cancer treatment in a rat model of mammary cancer

**DOI:** 10.1186/s12864-023-09423-0

**Published:** 2023-06-13

**Authors:** Anna Nynca, Sylwia Swigonska, Monika Ruszkowska, Agnieszka Sadowska, Karina Orlowska, Tomasz Molcan, Kamil Myszczynski, Iwona Otrocka-Domagala, Katarzyna Paździor-Czapula, Beata Kurowicka, Brian Kelli Petroff, Renata Elzbieta Ciereszko

**Affiliations:** 1 Department of Animal Anatomy and Physiology, University of Warmia and Mazury in Olsztyn, Oczapowskiego 1A, 10-719 Olsztyn, Poland; 2 Laboratory of Molecular Diagnostics, University of Warmia and Mazury in Olsztyn, Olsztyn, Poland; 3 Department of Human Nutrition, University of Warmia and Mazury in Olsztyn, Olsztyn, Poland; 4 Department of Reproductive Immunology and Pathology, Institute of Animal Reproduction and Food Research, Olsztyn, Poland; 5 Department of Biochemistry and Molecular Biology, Michigan State University, East Lansing, MI USA; 6 Laboratory of Translational Oncology, Intercollegiate Faculty of Biotechnology, University of Gdańsk and Medical University of Gdańsk, Gdansk, Poland; 7 Department of Pathological Anatomy, Faculty of Veterinary Medicine, University of Warmia and Mazury in Olsztyn, Olsztyn, Poland; 8 Department of Pathobiology and Diagnostic Investigation, Michigan State University, East Lansing, MI USA

**Keywords:** Tamoxifen, Cyclophosphamide, Fertility preservation, Tumor-bearing rats, Apoptosis, Transcriptome/proteome

## Abstract

**Background:**

Premenopausal women diagnosed with breast cancer often face aggressive chemotherapy resulting in infertility. Tamoxifen (TAM) is a selective estrogen receptor modulator that was previously suggested as a protective agent against chemotherapy-induced ovarian failure. In the current study, we examined mechanisms of the protective action of TAM in the ovaries of tumor-bearing rats treated with the chemotherapy drug cyclophosphamide (CPA).

**Results:**

TAM prevented CPA-induced loss of ovarian follicular reserves. The protective TAM effect in the rat ovary partially resulted from decreased apoptosis. In addition, transcriptomic and proteomic screening also implicated the importance of DNA repair pathways as well as cell adhesion and extracellular matrix remodeling in the protective ovarian actions of TAM.

**Conclusions:**

Tamoxifen shielded the ovary from the side effects of chemotherapy without lessening the tumoricidal actions of mammary cancer treatment.

**Graphical Abstract:**

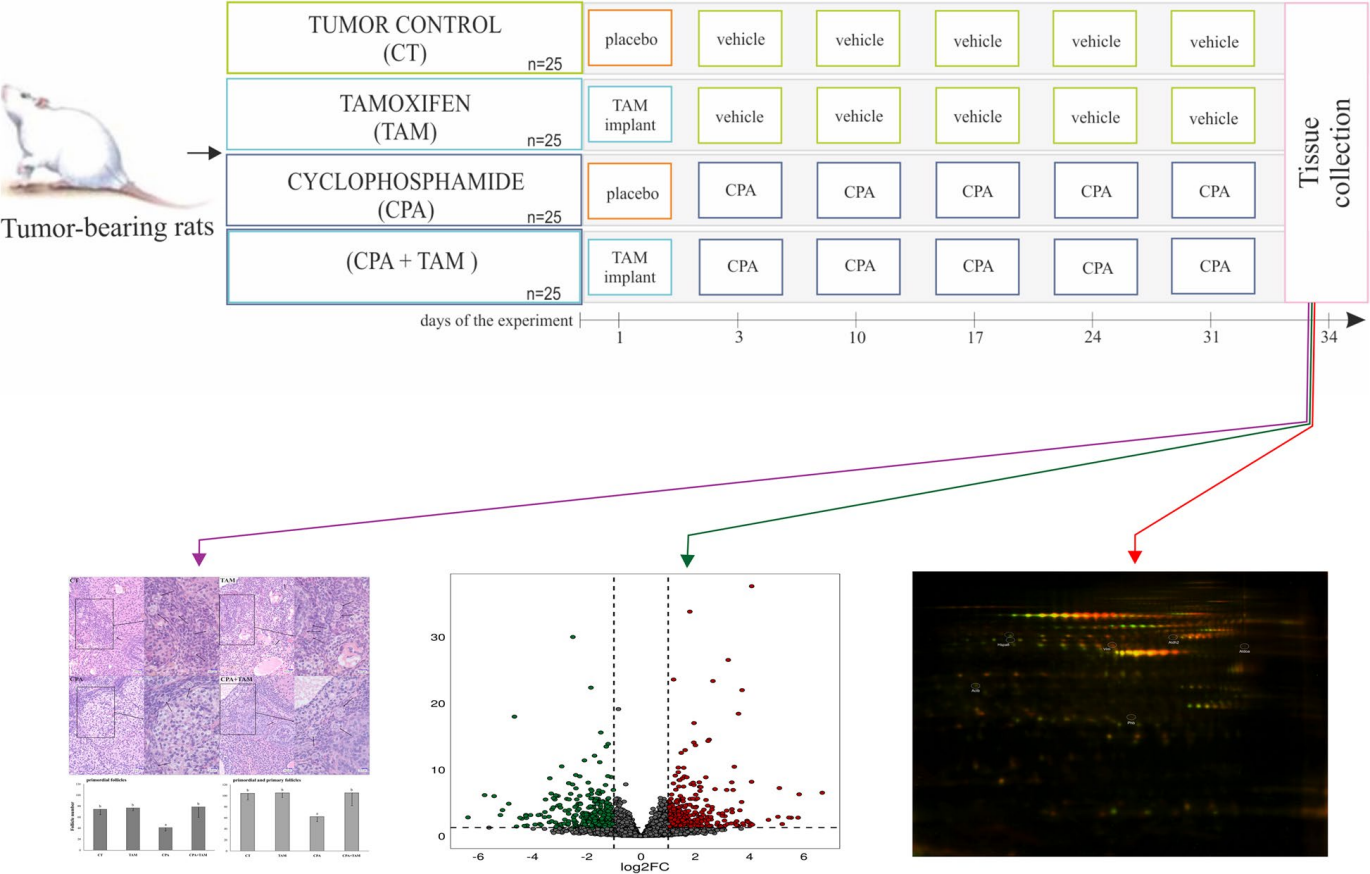

**Supplementary Information:**

The online version contains supplementary material available at 10.1186/s12864-023-09423-0.

## Background

Women diagnosed with breast cancer often face aggressive chemotherapy associated with a significant decrease in their ovarian follicular reserve [1]. Cancer chemotherapy was reported to induce ovarian failure by blocking cell division and inducing DNA damage, which might result in premature exhaustion of a pool of resting primordial follicles [1]. Cyclophosphamide (CPA) is one of the standard chemotherapeutics that are still in use for breast cancer treatment [2]. CPA has been shown to induce direct and indirect DNA-damage and/or cellular stress, which are often followed by apoptosis [3]. To prevent follicular depletion resulting in infertility of cancer patients, a number of studies have investigated different substances or strategies to protect the ovaries from chemotherapy-induced damage [3, 4].

Cryopreservation of embryos or oocytes is the only well-established method for fertility preservation in breast cancer patients [5, 6]. Nevertheless, cryopreserving oocytes/embryos does not protect against the risk of chemotherapy-induced ovarian damage [7]. Therefore, other strategies including hormonal protection were developed to protect ovaries of women treated for cancer. Results from the available randomized trials demonstrated that gonadotropin-releasing hormone (GnRH) analogs may have protective effects in reducing the risk of chemotherapy-induced ovarian failure [7, 8]. However, the mechanisms underlying their protective role of ovarian suppression during cancer treatment have not been fully recognized. Tamoxifen (TAM; a selective estrogen receptor modulator), which has so far been used mainly as an adjuvant therapeutic in breast cancer, in recent preclinical studies has shown the potential to alleviate ovarian side effects of cancer treatment [8–10]. Specifically, TAM blocked CPA-induced follicular toxicity in a rat model [8, 9]. Similar results were obtained in vitro where TAM reduced CPA-induced follicle loss in neonatal rat ovaries [10]. Although the latter study provided some limited data concerning processes affected by TAM (apoptosis, inflammation, tissue remodeling), the mechanism of the TAM action in the ovary has not been elucidated. In the current study, we employed transcriptomic and proteomic approaches to examine the potential molecular pathways underlying the protective TAM effects against the chemotherapy-induced toxicity comprehensively. Moreover, in contrast to previous experiments, the current study was performed on rats with mammary neoplasia. This approach assesses both the shielding effects of TAM on the ovary and the interaction of TAM with simultaneous chemotherapy of mammary cancer. This observes directly for the first time whether TAM compromises the effects of cancer treatment during fertility preservation. The results of our study will allow for a better understanding of the protective mechanism of TAM action in the ovary.

## Results

### Mammary tumor number and mammary gland histopathology in CPA and/or TAM-treated rats

Mammary gland tumors and/or pre-neoplastic lesions (epitheliosis and lobuloalveolar hyperplasia with atypia—data not shown) were present in all N-methyl-N-nitrosourea (MNU)-treated rats. Total body weight of all animals during the experiment is shown in Table S1. The number of tumor-bearing rats per group, the number of tumors found in each group as well as the incidence of MNU-induced tumors in all groups are presented in Table 1. The incidence of MNU-induced tumors was significantly lower in CPA-treated groups compared to control group (*p* < 0.05). Tamoxifen affected neither the incidence of tumors nor the tumor number (Table 1). In addition, TAM did not affect the incidence or the severity of atypia in the pre-neoplastic lesions detected in mammary glands (data not shown). Vehicle-treated rats (CNT group) displayed normal histology of mammary glands and did not have any tumors.

**Table 1:** N-methyl-N-nitrosourea (MNU)-induced tumor incidence in rats treated with tamoxifen (TAM) and/or cyclophosphamide (CPA) on day 34 of the experiment

	**CT**	**TAM**	**CPA**	**CPA + TAM**
number of tumor-bearing rats per group	11/25^b^	10/24^b^	3/23^a^	2 /22^a^
number of tumors (3–10 mm in diameter)	10	10	3	1
number of tumors (11–250 mm in diameter)	9	6	3	1
tumor incidence (%)	44.0	41.7	13.0	9.1

*CT* Control group

^a,b^: different superscripts depict significant differences between groups (*p* < 0.05)

### The follicle number and apoptosis rate in ovarian cells in tumor-bearing rats treated with CPA and/or TAM

**Fig. 1:**
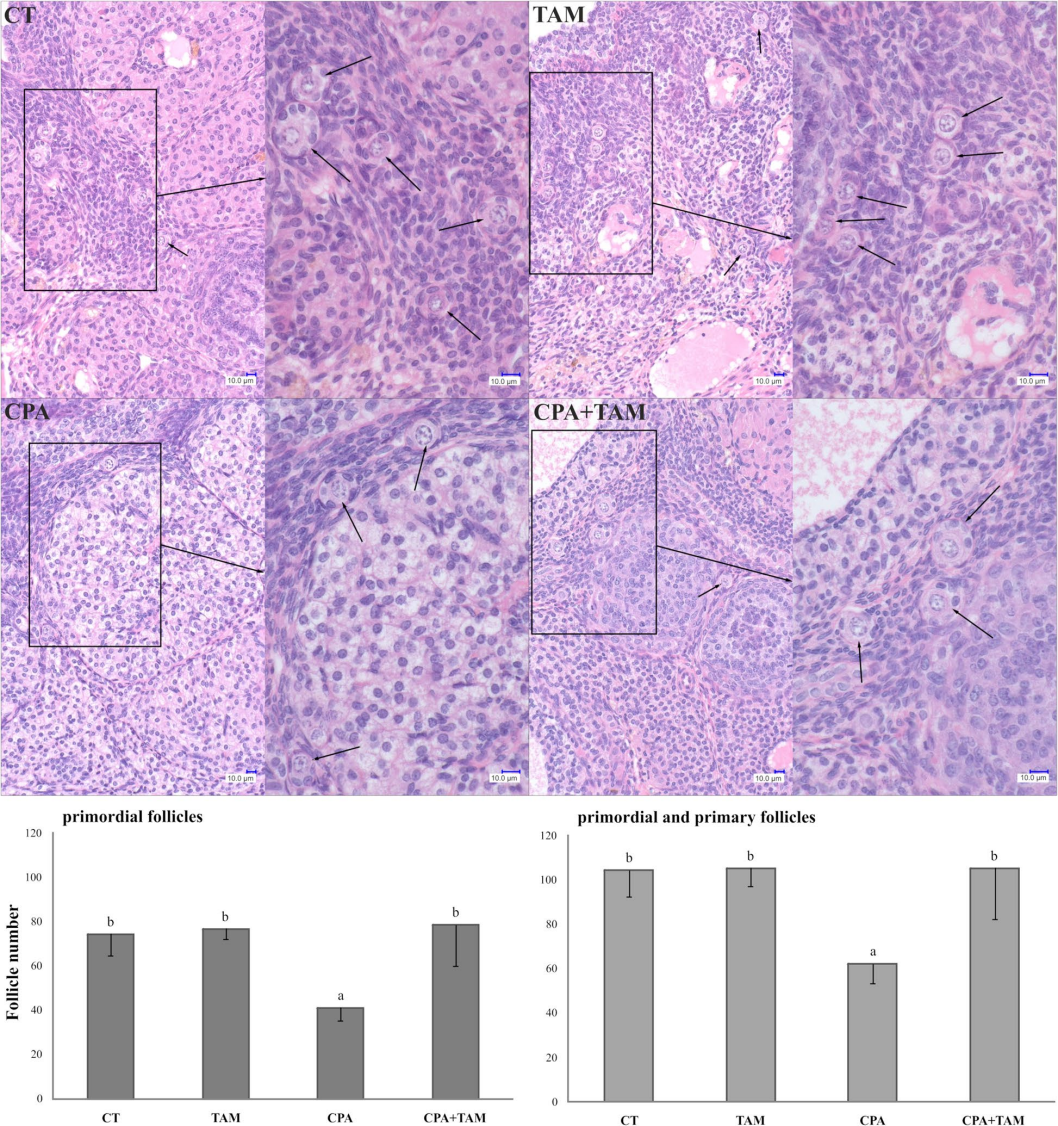
Upper panel: Representative images of hematoxylin–eosin stained ovarian sections obtained from tumor-bearing rats treated with vehicle (CT, control group), tamoxifen (TAM), cyclophosphamide (CPA) or CPA + TAM. Primordial follicles and primary follicles are marked by arrowheads. Scale bars are shown on each subfigure. Lower panel: The number of primordial follicles and primordial plus primary follicles in the ovaries of tumor-bearing rats treated with vehicle, TAM, CPA or CPA + TAM. Values are expressed as mean ± SEM of follicles counted per ovary (*n* = 5 ovaries/group; *p* < 0.05)

Cyclophosphamide treatment decreased (*p* < 0.05) the number of primordial and primordial plus primary follicles. TAM blocked this toxic effect of CPA in both types of follicles (Fig. 1). Tamoxifen alone, however, did not affect the number of follicles. Representative histological images of ovarian tissue from all experimental groups are presented in Fig. 1.

**Fig. 2:**
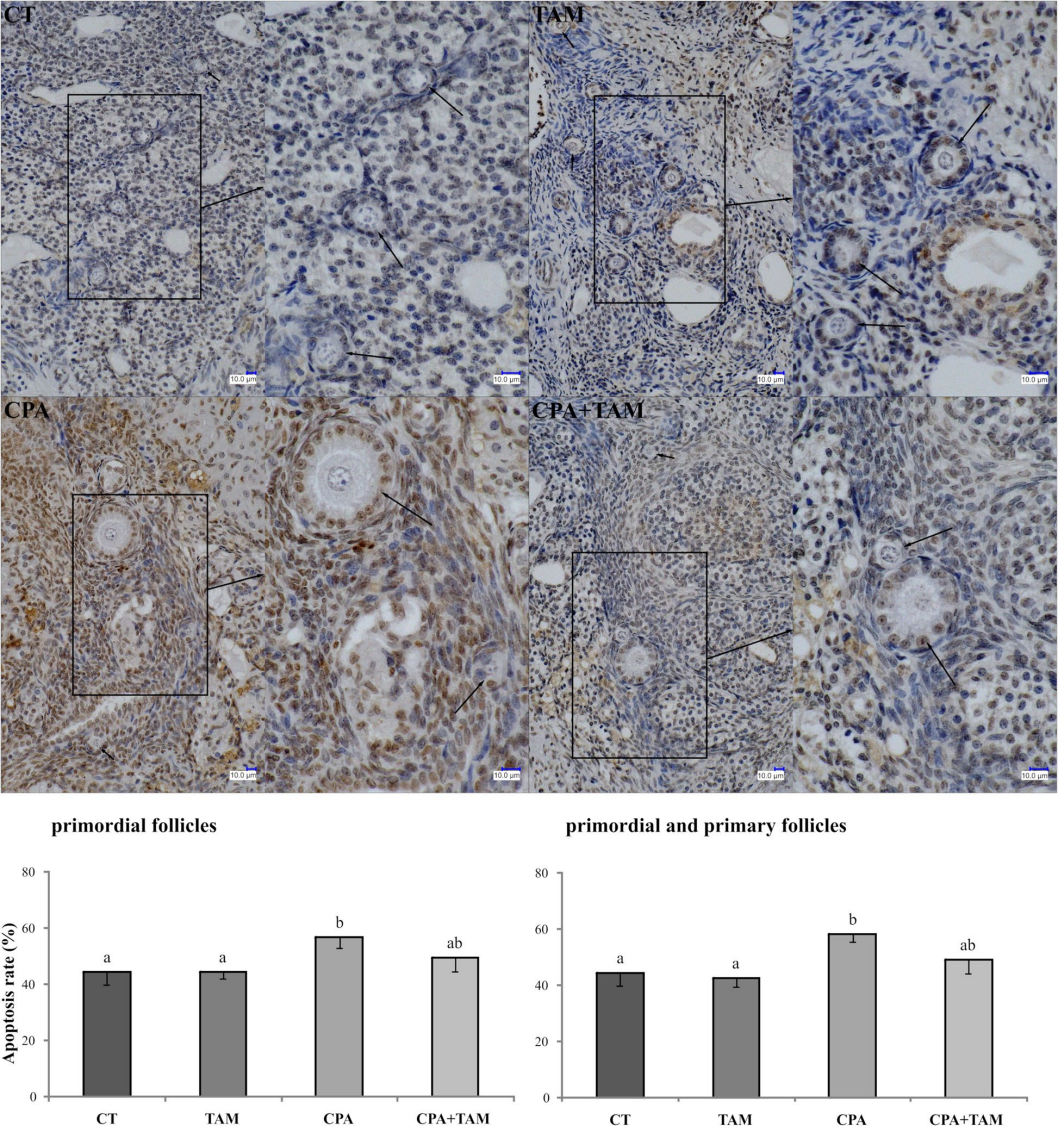
Upper panel: Representative images of TUNEL-stained ovarian sections obtained from tumor-bearing rats treated with vehicle (CT, control group), tamoxifen (TAM), cyclophosphamide (CPA) and CPA + TAM. The brown color indicates apoptotic cells. Scale bars are shown on each subfigure. Lower panel: The percentage of primordial and primordial plus primary follicles with apoptotic cells detected by TUNEL in the ovaries (*p* < 0.05) of tumor-bearing rats treated with vehicle, TAM, CPA or CPA + TAM. Values are expressed as mean ± SEM of follicles counted per ovary (*n* = 5 ovaries/group)

Cyclophosphamide increased the prevalence of apoptosis in the primordial and primary follicles remaining in the ovary (*p* < 0.05; Fig. 2). Tamoxifen tended to reverse the apoptotic effect of CPA (*p* = 0.12) in primordial plus primary follicles. However, TAM alone did not affect the apoptosis rate in follicular cells of rats. Representative terminal deoxynucleotidyl transferase dUTP nick end labeling (TUNEL) staining images of ovarian tissue from all experimental groups are presented in Fig. 2.

### Effect of TAM on the transcriptome of CPA-treated ovaries

**Fig. 3:**
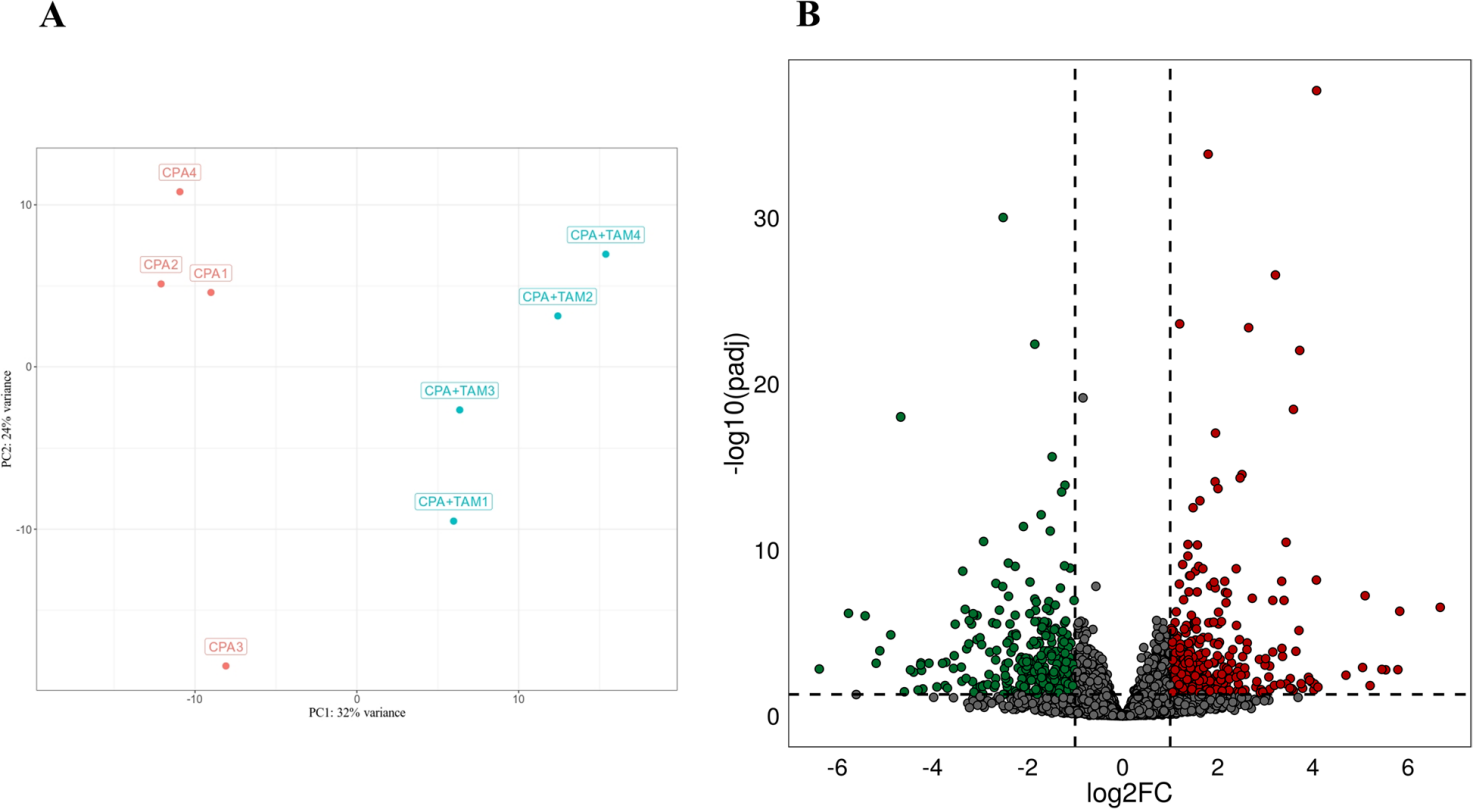
**A/** Graphical representation of the first (PC1) and second (PC2) principal components (PC) affecting the gene expression pattern of ovaries isolated from tumor-bearing rats treated with cyclophosphamide (*n* = 4; CPA1–4) or CPA plus tamoxifen (*n* = 4; CPA + TAM1-4). **B/** The volcano plot presents differentially expressed genes (DEGs; normalized counts, p-adjusted < 0.05 and log2 fold change [log2FC] ≥ 1.0 or log2FC ≤ -1.0) identified in the ovaries collected from rats treated with CPA plus TAM *vs*. rats treated with CPA alone. DEGs are represented by multicolored dots, where red color depicts up-regulated DEGs and green color down-regulated DEGs. The grey dots represent all genes that were identified in the ovaries

The sequencing data from the current study were submitted to the BioProject database under accession number: PRJNA640997. Sequencing of mRNA isolated from rat ovaries produced 68.6–86.0 million raw reads/sample. After removing reads containing adapters and low-quality reads (reads length < 50 bp; Phred score Q < 20), the remaining high-quality reads were mapped to the Ensembl rat genome (*Rattus norvegicus* version 6.0). The number of reads aligned to the reference genome ranged from 63.5 to 80.4 million per sample, and an average of 89.7% of the reads were mapped to unique locations. The total number of genes expressed in ovaries of tumor-bearing rats of all examined samples ranged from 19,803 to 20,339 (Table S2). The results of the Principal Component Analysis (PCA) revealed a high level of similarity between the biological replicates within each particular rat group (Fig. 3A). The Volcano plot presents the significant differences (P-adjusted < 0.05, log2FC ≥ 1.0 or ≤ -1.0) in gene expression profiles of the ovaries collected from tumor-bearing rats treated with CPA + TAM in comparison to rats treated with CPA alone (Fig. 3B).

**Fig. 4:**
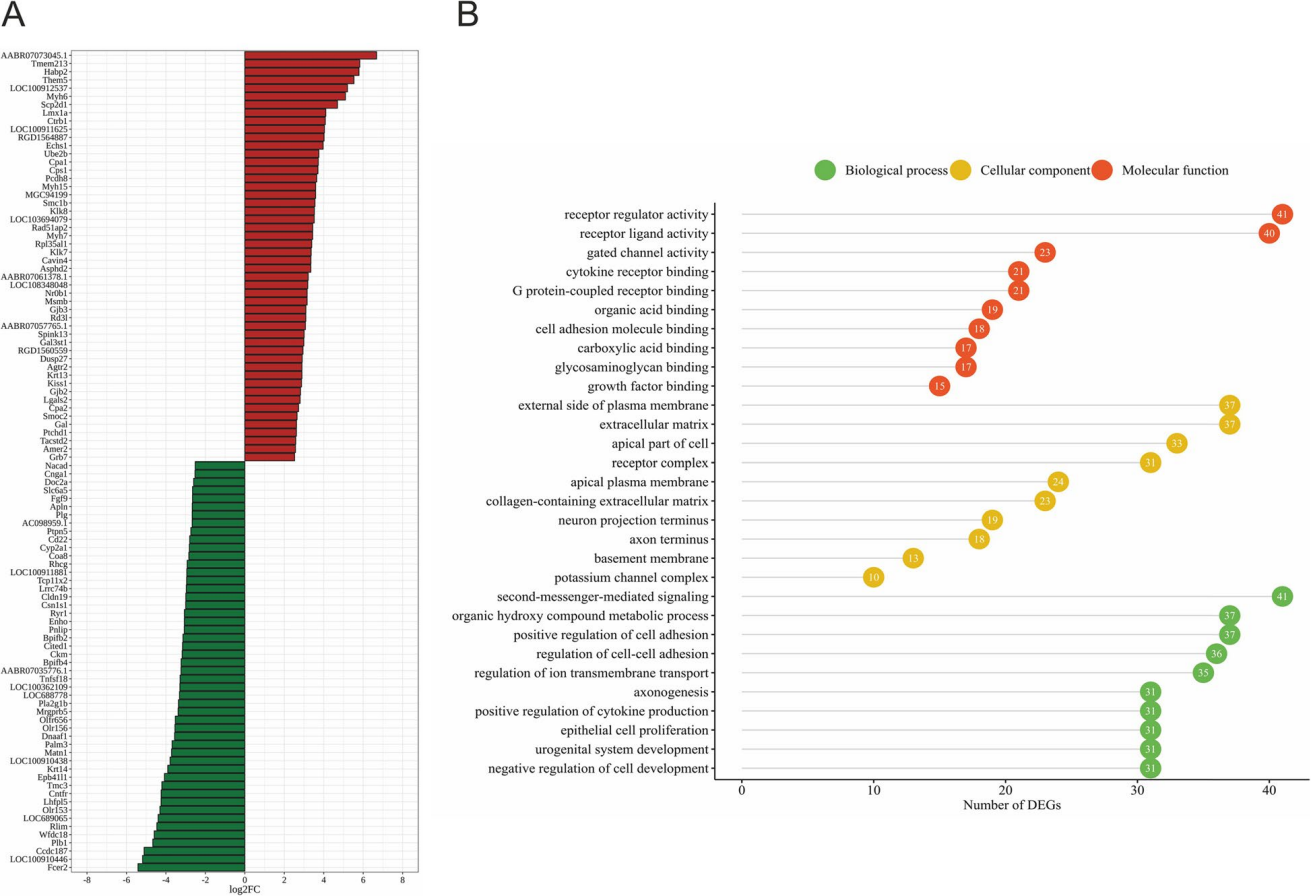
**A/** Heatmap of the top 100 differentially expressed genes (DEGs) demonstrated in the ovaries collected from rats treated with cyclophosphamide (CPA) plus tamoxifen (TAM) *vs*. rats treated with CPA alone. The results were considered statistically significant at p-adjusted < 0.05 and log2 fold change (log2FC) ≥ 1.0 or log2FC ≤ -1.0. Red blocks represent up- and green blocks down-regulated genes. **B/** Gene Ontology (GO) analysis of differentially expressed genes (DEGs) identified in the ovaries collected from rats treated with CPA plus TAM *vs*. rats treated with CPA alone. 702 out of 770 DEGs were classified into three categories of the GO database (''biological processes'', ''cellular components'' and ''molecular function''). The number of the DEGs ascribed to the particular GO term is presented in circles

A total of 770 differentially expressed genes (DEGs) were determined in the current study (Table S3). We identified 334 down- and 436 up-regulated DEGs in rat ovaries of the CPA + TAM group compared to those of the CPA group. The expression profiles of top 50 up- and top 50 down-regulated DEGs (i.e., DEGs with the highest log2FC values) are presented in Fig. 4. The log2FC values for DEGs ranged from -5.41 (Fcer2, Fc fragment of IgE receptor II) to 6.68 (AABR07073045.1, 40S ribosomal protein S25-like) (Table S3).

### Functional enrichment of the identified DEGs

To examine the possible significance of the identified DEGs in the ovaries collected from CPA + TAM-treated rats in comparison to CPA-treated rats, the genes were assigned to three main categories of GO database (“biological processes” [BP], “cellular components” [CC], “molecular function” [MF]). Seven hundred two out of 770 DEGs were ascribed to 259 GO terms (P-adjusted < 0.05) including 220 terms within BP, 15 terms within CC and 24 terms within MF categories (Fig. 4; Tab. S4). Within the BP category, the DEGs were enriched mainly in “second-messenger-mediated signaling”, “regulation of cell–cell adhesion”, “positive regulation of cell adhesion”, “hormone secretion” and “hormone transport”. Within the CC category, the most DEGs were assigned to “extracellular matrix”, “external side of plasma membrane” and “collagen-containing extracellular matrix”. Within the MF category, the DEGs were ascribed mainly to “receptor ligand activity”, “receptor regulator activity”, “cell adhesion molecule binding” and “glycosaminoglycan binding” (Tab. S4).

**Fig. 5:**
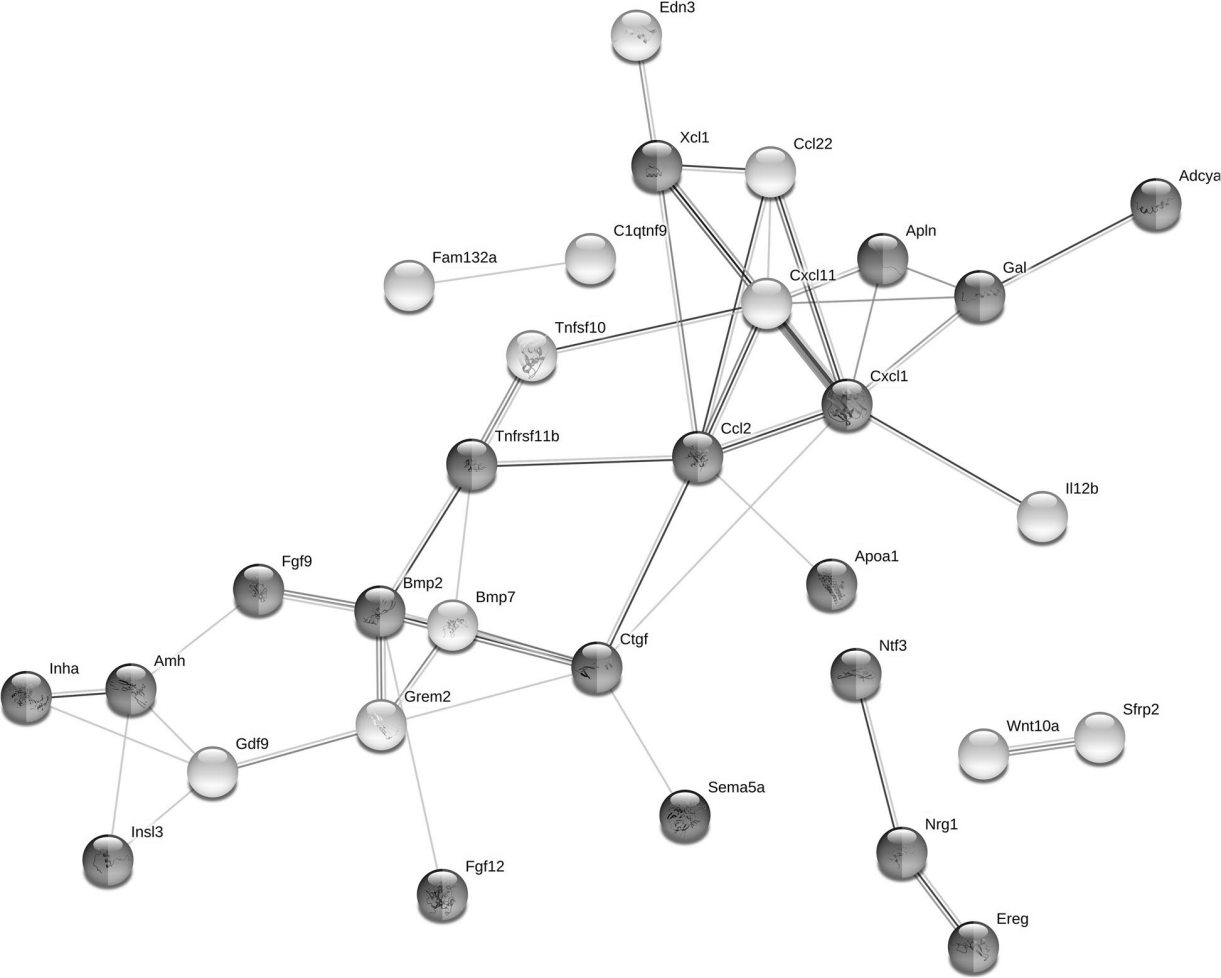
Interaction network of differentially expressed genes (DEGs) identified in the ovaries collected from rats treated with cyclophosphamide (CPA) plus tamoxifen *vs*. rats treated with CPA alone. The network was generated by STRING (confidence score: 0.4) using DEGs (P-adjusted < 0.05 and log2 fold change ≥ 1.0) belonging to the GO “receptor regulator activity” term (GO:0030545). Enrichment *P*-value: 1.0 × 10^–16^

The “receptor regulator activity” was one of the most enriched GO term containing 41 DEGs. Functional classifications of these genes performed with the use of STRING produced a gene interaction network with 41 nodes and 46 edges (protein–protein interaction enrichment P-value: 1.0 × 10^–16^; Fig. 5). The network includes genes related to the regulation of signaling receptor activity and regulation of cell communication (anti-mullerian hormone [Amh], inhibin subunit alpha [Inha], insulin-like 3 [Insl3], tumor necrosis factor receptor superfamily member 11B [Tnfrsf11b], fibroblast growth factor 9 and 12 [Fgf9, Fgf12], bone morphogenetic protein 2 [Bmp2], connective tissue growth factor [Ctgf] and epiregulin [Ereg]). We also identified a group of DEGs which have been shown to be associated with apoptosis (e.g. NRG1, ErbB2, TCTN3, Wfdc18) and DNA damage (TP63, Il-12, Tnfrsf11b, Cdkn1c).

### Validation of RNA-Seq data by real-time PCR

To validate the RNA-Seq results, four DEGs were randomly chosen for real-time PCR analysis (Tab. S5). Klk7 (log2FC: 3.36) and Amh (log2FC: 1.43) were up-regulated, while Pla2g1b (log2FC: -3.36) and Igf1r (log2FC: -1.03) were down-regulated. The expression patterns of the four selected DEGs obtained by real-time PCR entirely confirmed the results obtained by RNA-Seq (Fig. S1).

### The ovarian proteome of tumor-bearing rats treated with CPA and/or TAM

A DIGE-based proteomic approach was used to identify differentially expressed protein spots (DEPSs) in the ovaries of tumor-bearing rats treated with CPA + TAM or CPA alone. A total of 959 protein spots were detected on all gels, and 578 of the protein spots were successfully matched between the gels obtained from CPA + TAM and CPA ovaries. Representative gel is presented in Figure S2. Within these spots, the abundance of seven spots (DEPSs) significantly differed (*p* < 0.05; fold change > 1.5) between the compared groups of rats. The proteins were submitted to MALDI TOF/TOF MS analysis and six of them were identified as vimentin (Vim), prohibitin (Phb), heat shock cognate 71 kDa protein (Hspa8), mitochondrial aldehyde dehydrogenase (Aldh2), fructose-bisphosphate aldolase A (Aldoa) and cytoplasmic actin 1 (Actb; Tab. 2). Some of these proteins were shown to be related to apoptosis and DNA damage (Aldh2, Hspa8, Aldoa, Phb).

**Table 2:** Differentially expressed proteins identified in the ovaries of rats treated with cyclophosphamide plus tamoxifen *vs*. rats treated with cyclophosphamide

Identified proteins	MASCOT protein score	Sequence coverage [%]	Number of peptides	Fold change	Accession number
Vimentin	287	26	12	-1.8	gi ǀ38197662
Prohibitin	83	21	5	-1.6	gi ǀ13937353
Fructose-bisphosphate aldolase A	108	16	4	-2.4	gi ǀ408772019
Heat shock cognate 71 kDa protein	107	10	5	2.2	gi ǀ13242237
Aldehyde dehydrogenase, mitochondrial	263	16	8	1.7	gi ǀ1820958497
Actin, cytoplasmic 1	105	35	1	2.1	gi ǀ13592133

## Discussion

In the present study we examined mechanisms of the protective action of TAM in the ovaries of rats treated with the widely used chemotherapy drug cyclophosphamide (CPA). Cyclophosphamide decreased the ovarian follicular reserve, in part by inducing apoptosis in follicular cells. TAM prevented the follicular loss caused by CPA in agreement with previous studies [9–11]. TAM alone did not alter the follicle number or prevalence of granulosal apoptosis. Unlike the previous studies [9, 10], these findings were demonstrated in rats bearing mammary tumors. This allowed us to show that TAM protected the ovary from toxicity in this model without lessening the tumoricidal actions of cancer treatment. The changes induced by TAM in the ovarian transcriptomes and proteomes were consistent and implied that activation of anti-apoptotic pathways may participate in the protective actions of TAM in the ovary. Moreover, the identified DEGs and DEPs strongly suggest the involvement of DNA repair pathways and processes associated with cell adhesion and extracellular matrix (ECM) remodeling in these actions.

We found that the decrease in follicle number observed in rats being treated for mammary cancer was prevented by TAM. Previous in vivo work performed using cancer-free rats also demonstrated the protective effects of TAM against CPA or 7,12-dimethylbenz[α]anthracene (DMBA)-induced ovarian toxicity [9]. Similarly, TAM blocked the toxic effect of CPA on cultured rat ovaries [10]. Another group of researchers demonstrated protective effects of TAM against radiotherapy-induced ovarian failure in cancer-free rats [12]. Infertility from cancer treatment is thought to be due in large part to premature menopause following depletion of ovarian follicles [4, 13]. The shielding effect of TAM in this and previous studies appears to predominantly benefit the small pre-antral follicles that constitute the majority of the ovarian reserve. Additionally, the protective ovarian actions of TAM do not appear to involve increased metabolism of CPA since its tumoricidal actions were undiminished. Furthermore, past studies documented a similar direct blockade of ovarian toxicity from active metabolites of CPA and doxorubicin (as well as the experimental toxicant dimethylbenzanthracene) in vitro [9].

Tamoxifen binds to estrogen receptors (ERs) and elicits estrogen agonist or antagonist responses in a tissue specific manner [14]. In breast tissue, TAM acts predominantly as an estrogen antagonist, and as such is a part of a standard therapy for treating estrogen receptor-positive breast cancers [11, 15, 16]. In the ovary, TAM action seems to depend on the concentrations of endogenous estrogens and estrogen response element cofactors as well as the abundance of ER isoforms [17]. TAM was found to increase the number of primordial follicles in the ovaries of healthy mice [18], which supports the thesis of a protective effect on the ovarian reserve. TAM was also reported to increase the number of large atretic follicles in rats [19]. In women, TAM can promote transient formation of ovarian cysts in breast cancer patients after mastectomy and/or chemotherapy [20, 21]. Primordial and primary follicles were not examined in these studies.

The mechanisms of TAM action in cancer cells have been widely studied, mostly in the context of estrogen receptor-positive breast cancer treatment [22, 23]. However, only a few researchers dealt with TAM and the ovary during chemotherapy [9, 10, 12]. In the current study, CPA treatment resulted in a significant increase in the apoptosis rate of cells forming primordial and primary ovarian follicles of tumor-bearing rats. This is consistent with previous studies showing that CPA induced apoptosis within ovarian follicles of rats [10] and mice [24–26]. Moreover, we found that TAM tended to reverse the apoptotic effect of CPA on primordial and primary follicles and activated anti-apoptotic target genes. Similar effects were observed in a previous in vitro study, where TAM significantly reversed CPA-induced apoptosis in primordial and primary follicles [10] and TAM inhibited doxorubicin-induced oocyte fragmentation [9]. TAM also prevented radiotherapy-induced apoptosis in rat follicles [12]. These findings support the notion that TAM has tissue specific anti-apoptotic properties that may protect ovaries from side effects of chemotherapy or radiation.

To further explore the protective role of TAM, RNA-Seq was used to examine the TAM-induced changes in the transcriptome of ovaries harvested from tumor-bearing rats treated with CPA. A total of 770 DEGs were determined with 334 genes being down- and 436 being up-regulated. Among these DEGs, we identified an array of genes that are known to be involved in the regulation of apoptosis. For example, neuregulin-1 (NRG1) and Erb-B2 receptor tyrosine kinase 2 (ErbB2) were demonstrated to inhibit apoptosis of granulosa cells in rats [27]. Overexpression of ErbB2 in Chinese hamster ovarian cells stably expressing the anti-apoptotic gene Bcl-x_L_ suggested its pro-survival properties [28]. In addition, an activated ErbB2 inhibited apoptosis and induced proliferation in mammary epithelial cells [29]. In cervical cancer, in turn, the up-regulated expression of ErbB2 was linked to enhanced proliferation and migration of cancer cells [30]. On the other hand, the inhibition of ErbB2 expression in different ovarian cancer cell lines resulted in a significant increase of apoptosis evidenced by changes in caspase activity [31]. In the present study, TAM significantly increased the ovarian expression of NRG1 and ErbB2, genes with anti-apoptotic effects.

Tectonic family member 3 (TCTN3) and WAP four-disulfide core domain 18 (Wfdc18, Expi) are another pair of apoptosis-associated DEGs identified in the present study. TAM greatly up-regulated (FC: 5.2) the expression of TCTN3 in the ovaries of tumor-bearing and CPA-treated rats. The loss of the expression of TCTN3 caused neuronal apoptosis in mice [32]. The expression of Wfdc18, in turn, was markedly down-regulated (FC: -4.6) by TAM in our study. The over-expression of Wfdc18 in mammary epithelial cells accelerated apoptosis by inducing the expression of several genes linked to apoptosis [33]. Although we did not find a direct connection between TAM and Bax, Bcl-2 or caspases (the best known genes involved in the regulation of apoptosis), we found that TAM changed the expression of a number of genes connected to apoptosis (Tables S3, S4). However, the actual significance of these changes and their interrelationships are still obscure.

Cells exposed to cytotoxic conditions or substances (e.g., CPA) activate DNA repair pathways. If repair is not possible they initiate cell death pathways. In the current study, TAM affected not only the expression of apoptosis-related genes, but also the expression of those responsible for DNA-repair. We found that the ovarian expression of TP63 was higher in (CPA + TAM)-treated rats compared to CPA group (FC: 1.6). The TP63 belongs to p53 family of transcription factors, which play a critical role in the apoptotic response to DNA damage caused by chemotherapeutics. TP63 was activated in mice oocytes in response to DNA damage [34]. On the other hand, siRNA knock-down of TP63 expression resulted in the repression of DNA damage repair genes and the activation of pro-apoptotic genes in keratinocytes [35]. Interleukin 12 (Il-12), expression of which was increased by TAM in our study (FC:2.0), was also found to regulate DNA repair processes. Specifically, Il-12 inhibited UVB-induced apoptosis of keratinocytes by inducing nucleotide-excision repair [36]. Other DEGs that may be involved in DNA repair include among others: Rad51ap2, Cdkn1c, Smc1b, Slx4ip and Myh6. It seems that TAM can also initiate mechanisms that protect follicles from CPA-induced toxicity by influencing the signaling of DNA damage repair.

Proteomic analysis allowed us to identify six of the seven proteins with abundances that were significantly different between ovaries of (CPA + TAM)- and CPA-treated rats. TAM down-regulated the abundance of vimentin (Vim), prohibitin (Phb) and fructose-bisphosphate aldolase A (Aldoa) proteins and up-regulated heat shock cognate 71 kDa protein (Hspa8), mitochondrial aldehyde dehydrogenase (Aldh2) and cytoplasmic actin 1 (Actb). Similar to the transcriptomic data, several of the proteins altered by TAM are involved in apoptosis and/or DNA repair. For example, Aldh2 reduced apoptosis in human peripheral blood mononuclear cells [37]. Zhai et al*.* [38] found that Aldh2 attenuated oxidative stress and detoxified reactive aldehydes in CPA-induced hepatotoxicity in mice. Moreover, Hsp70 proteins, including Hspa8, also have anti-apoptotic properties (for review see: [39]). On the other hand, a glycolytic enzyme protein, Aldoa, was down-regulated by TAM. Overexpression of Aldoa induced p53-dependent apoptosis in xenograft tumors in mice [40]. In pancreatic cancer cells, Aldoa inhibited DNA repair and in consequence promoted cancer development [41]. The hypothesis that anti-apoptotic mechanisms and those related to DNA repair are involved in TAM action in rat ovaries during the CPA treatment seems to be justified.

In contrast to the other proteins down-regulated by TAM, Phb is often considered an anti-apoptotic factor. Its anti-apoptotic properties were demonstrated in granulosa and theca cells of immature rats [42, 43] and some cancer cells [44, 45]. No consensus exists on the role of Phb in apoptosis since its action appears to depend on cell type, age and differentiation status, stage of follicular development and gonadotropin stimulation as well as the concentration and subcellular localization of Phb [42, 46, 47]. The mechanism of Phb action in the ovaries of tumor-bearing mature rats requires further study.

In summary, we have shown that TAM prevented the incidental loss of ovarian follicular reserves in rats treated with CPA for mammary neoplasia. TUNEL data indicated that the protective TAM effect in the rat ovary results in part from decreased apoptosis. In addition to apoptosis-related genes and proteins, transcriptomic and proteomic screening also implicated the importance of DNA repair pathways as well as cell adhesion and ECM remodeling in the protective actions of TAM in the ovary. The protective actions of TAM for the ovarian follicular reserve did not interfere with effects of the chemotherapy treatment against mammary tumors.

## Conclusions

Although simple extrapolation of these data to human tumorigenesis and cancer treatment is not possible, the results of this study provide additional support for the exploration of tamoxifen and its ability to preserve fertility and normal ovarian function in premenopausal women undergoing cancer chemotherapy. Regardless of the interesting results obtained with the use of “omics” methods in the current study, further research on the protective mechanism of tamoxifen in the ovary during chemotherapy requires in-depth functional studies.

## Methods

### Animals and treatments

**Fig. 6:**
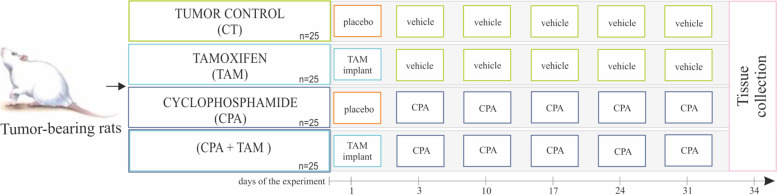
Experimental design. Placebo: empty implant; vehicle: 0.9% NaCl; CPA was injected intraperitoneally

All procedures involving rats were approved by the Animal Ethics Committee of the University of Warmia and Mazury in Olsztyn, Poland (No. 78/2017/WNP). All experiments were carried out in accord with the guide lines for the care and use of laboratory animals. Female Wistar rats (6 weeks old, *n* = 125) [48] were housed in a controlled environment (22 °C; 60% humidity; 12L:12D) in the Center of Experimental Medicine (Bialystok, Poland), with ad libitum access to food and water. To induce mammary gland tumors, N-methyl-N-nitrosourea (MNU; Toronto Research Chemicals, Canada; 50 mg/kg b.w.; in 0.9% NaCl plus 0.05% acetic acid) was administered (ip) twice to 100 rats, at 7 and 19 weeks of age [48]. The remaining 25 rats, which constituted a non-tumor control group (CNT), received vehicle (0.9% NaCl plus 0.05% acetic acid) at these times. At 31 weeks of age, the MNU-treated rats (*n* = 100), hereafter called tumor-bearing rats, were randomly assigned (simple randomization) to the four following groups (*n* = 25/group): 1/ control group (CT, tumor control group), 2/ tamoxifen (TAM)-treated group, 3/ cyclophosphamide (CPA)-treated group and 4/ (CPA + TAM)-treated group (Fig. 6). CPA is one of the standard chemotherapeutics used for breast cancer treatment [2]. On day 1 of the experiment, TAM rats received subcutaneous implants gradually releasing tamoxifen (1 mg/kg b.w./day; Innovative Research of America, Sarasota, USA) [49, 50]. On day 3, CPA rats were injected (ip) with 50 mg/kg b.w. of CPA at approx. 10 am (Sigma, St. Louis, USA; in 0.9% NaCl) followed by weekly injections (ip) of 10 mg/kg b.w. of CPA (days 10, 17, 24 and 31, at approx. 10 am) [51]. The CPA + TAM rats received both drugs in the manner described for TAM and CPA rats. The CT rats received placebo implants (day 1) and were treated with 0.9% NaCl (vehicle) on days corresponding to those of the CPA treatment (Fig. 6). Six rats were excluded from the experiment due to medical indications. All the remaining rats were sacrificed on day 34 of the experiment. Anaesthesia was induced by the administration of 4% isoflurane in medical oxygen. Rats were sacrificed by total bleeding from the heart. Tissue samples were collected and the animals were checked for tumors. Tumor incidence was compared by chi-square analysis (Statistica 13.3 StatSoft Inc., Tulsa, OH, USA). Differences with probability of *p* < 0.05 were considered statistically significant.

Ovaries were snap frozen in liquid nitrogen and stored in -80 °C (RNA-Seq, RT-PCR, 2D-DIGE) or placed in Bouin’s solution (histological examination, TUNEL). Sample size was determined by the technical requirements of the methods used in the experiment (n = 4 for RNA-Seq, RT-PCR; *n* = 5 for histological examination, TUNEL; n = 6 for 2D-DIGE). To ensure randomization of sampling, ovaries (left or right) for each end point were chosen arbitrarily. Mammary glands (*n* = 22–25/group) were collected, fixed in formalin, then embedded in paraffin and sectioned. The sections were stained with H&E and examined by a board certified veterinary pathologist.

### Ovarian histopathology

To investigate the ovarian follicle counts, ovaries were harvested on day 34 of the experiment (*n* = 5/group). Ovarian follicle counts [52] were used as a direct marker of follicular reserve of females treated with TAM and/or CPA. The ovarian tissues were first fixed in Bouin’s solution, were then embedded in paraffin, sectioned (5 μm sections) and stained with H&E. The number of follicles at each developmental stage was counted in 8–10 separate sections – selected from central and different sagittal sections – per ovary. The examined sections were separated by 20 μm distance, preventing overlapping. In addition, to avoid double counting, only follicles with clearly visible oocyte nuclei were counted*.* The follicles were classified as: 1) primordial follicles, where oocyte is surrounded by a single layer of flattened granulosa cells, 2) primary follicles, where oocyte is surrounded by a single layer of cuboidal granulosa cells, 3) secondary follicles, where oocyte is surrounded by more than one layer of cuboidal granulosa cells, and 4) antral follicles characterized by a visible antrum. The follicles were counted by two independent researchers blinded to treatment group, and inter-observer concordance was above 90%. All sections were examined (200 × magnification) with a light microscope (Nikon Eclipse) and archived. One-way ANOVA followed by the least significant difference post hoc test were used for analysis of follicle numbers (Statistica 13.3 StatSoft Inc., Tulsa, OH, USA). Differences with probability of *p* < 0.05 were considered statistically significant.

### TUNEL staining

To detect follicular apoptotic cells and calculate the apoptosis rate in ovarian follicles, the ovaries were harvested on day 34 of the experiment (*n* = 5/group) and ovarian sections were prepared as described in Sect. 2.2. TUNEL staining was performed using the ApopTag Plus Peroxidase In Situ Apoptosis Detection Kit (EMD Millipore Corporation, USA). TUNEL-positive cells (granulosa cells and oocyte) were visualized and counted in 8–10 separate sections (selected from central and different lateral sections) per ovary. The examination was performed by two researchers (blinded to treatment group) using a Nikon Eclipse microscope (Japan, magnification 200x), and an inter-observer concordance was above 90%. One-way ANOVA followed by the least significant difference post hoc test was used for data analysis (Statistica 13.3 StatSoft Inc., Tulsa, OH, USA). The apoptosis rate was defined as the ratio of the number of apoptotic cells to the total number of cells. Differences with probability of *p* < 0.05 were considered statistically significant.

### Total RNA isolation and sequencing

To examine changes in transcriptome profiles total RNA was isolated from ovaries (*n* = 4 rats/group) using peqGold TriFast reagent. RNA concentration and quality were determined spectrophotometrically (NanoVue Plus, GE Healthcare, Little Chalfont, UK). To evaluate RNA integrity, a microfluidic electrophoresis (2100 Bioanalyzer; Agilent Technologies, Santa Clara, CA, USA) was employed. The samples with RNA integrity number (28 S/18 S ratio) at least 8.0 were used for RNA-Seq performed by Macrogen (Seoul, South Korea). Total RNA was used to construct cDNA libraries (TruSeq stranded mRNA Sample Preparation Kit, Illumina, San Diego, USA). The libraries were prepared by random fragmentation of cDNA samples followed by 5` and 3` adapter ligation. Adapter-ligated fragments were amplified (PCR). The cDNA library templates were then loaded into the flow cells where fragments were captured on a lawn of surface-bound oligos complementary to the library adapters. Each fragment was then amplified into distinct, clonal clusters through bridge amplification. After cluster generation was complete, the cDNA templates were designated for sequencing. A NovaSeq6000 high throughput sequencing instrument (Illumina) was used for 100 bp paired-end configuration sequencing.

### Bioinformatic analysis of gene expression

The quality of cDNA fragments obtained after sequencing (raw reads) was first evaluated using FastQC program (http://www.bioinformatics.babraham.ac.uk/projects/fastqc/). Raw reads were then trimmed by removing the adapter sequences, reads shorter than 50 bp and low quality reads (Trimmomatic tool version 0.39) [53]. After trimming, the reads were mapped to the reference genome of the rat (*Rattus norvegicus* version 6.0; Ensembl database release 99) with the use of HISAT2 software (version 2.1.0) [54]. The mapped reads were assembled into transcripts with StringTie tool (version 2.0) [55]. Differentially expressed genes (DEGs) were identified using DESeq2 package (version 1.26.0) with the R software (version 3.6.2) [56]. The calculated p-values were adjusted using Benjamini–Hochberg correction [57]. The threshold used to define the significant difference in gene expression was set at P-adjusted ≤ 0.05 and absolute value of log2 fold change (log2FC) ≥ 1. In order to assess the variability of the dataset, Principal Component Analysis (PCA) on four biological replicates of ovarian samples collected from CPA- and (CPA + TAM)-treated rats was performed using logarithmic normalized counts and R package. The figures presenting transcriptomic data were generated by R software using ggplot2 (version 3.2.1) [58] and gplots (version 3.0.1.1) [59] packages.

### Functional enrichment analysis (GO and STRING pathways)

Functional analysis of the identified DEGs was performed based on the Gene Ontology (GO) database, using clusterProfiler (version 3.14.1) [60], DOSE (version 3.12.0) [61], biomaRt (version 2.42.0) [62] and org.Rn.eg.db (version 3.10.0) [63] packages of R software, with the established criterion *P*-adjusted ≤ 0.05. Figure presenting GO data was generated by R software using ggplot2 and pathview (version 1.26.0) [64] packages. The Bioinformatics Database STRING 10.5 (Search Tool for the Retrieval of Interacting Genes, http://string-db.org) was used to investigate possible association networks between the identified DEGs [65]. The searching criteria were based on the occurrence of genes/proteins in scientific texts (text mining), co-expression and experimentally observed interactions. This analysis generated gene/protein interaction networks, where the strength of the interaction score was set as 0.4.

### Real-time PCR

Real-time PCR was used to validate the results of RNA-Seq by measuring the expression of four randomly selected DEGs identified in the ovaries of tumor-bearing rats treated with CPA + TAM in comparison to those of rats treated with CPA alone (*n* = 4 rats/group). The RT reaction and real-time PCR were performed as previously reported [66, 67]. Test t was used to analyze the DEG expression between groups (*p* < 0.05; Statistica 13.3 StatSoft Inc.) Primers and probes (Thermofisher Scientific, Waltham, MA, USA) for particular genes are presented in Table S5.

### Protein isolation and 2D-DIGE

To examine changes in proteome profiles, 9 rat ovarian proteins (*n* = 6 rats/group) were extracted with lysis buffer (7 M urea, 2% w/v CHAPS, 2% ampholytes [pH 4–7 NL; GE Healthcare, Chicago, IL, USA], 120 mM dithiothreitol, protease inhibitors cocktail [Sigma Aldrich], 0.002% bromophenol blue). The isolation and purification procedures were performed as previously described [68]. The protein concentration was determined before and after purification, using an adaptation of the Bradford assay [69] with bovine serum albumin (BSA) dissolved in rehydration buffer (7 M urea, 2 M thiourea, 2% CHAPS, 130 mM DTT, 2% ampholytes [pH 4–7 NL]) as a protein standard. BSA standards and the examined samples were acidified with 10 μl of 0.1 M HCl. The measurements were carried out at a wavelength of 595 nm using an Infinite M200 multimode microplate reader (Tecan, Grodig, Austria). The obtained protein extracts were used in 2D-DIGE.

The protein extracts (50 μg) from each sample (*n* = 6 ovaries/group) were dissolved in labeling buffer (30 mM Tris, 7 M urea, 2 M thiourea, 4% w/v CHAPS, pH 8.0) and labelled with CyDye DIGE Fluor minimal dyes (GE Healthcare, reconstituted in fresh 99.8% anhydrous dimethylformamide) at concentration of 400 pmol dye/50 μg of protein. The labeling was performed in the dark to avoid photobleaching of the fluorescent dyes (30 min, on ice). Differentially labeled proteins (Cy2-, Cy3-, Cy5-labeled) were mixed together. A dye swap of CPA- and (CPA + TAM)-treated samples was performed to exclude dye bias. The rehydration and separation by isoelectric focusing were performed as previously described [68]. The second dimension (SDS-PAGE) was performed using 12.5% SDS polyacrylamide gels in the Ettan DALTsix electrophoretic unit (GE Healthcare) at 20°C (1.5 W/gel for 16 h). To visualize the spots, the gels with separated proteins were scanned with an Ettan DIGE Imager (GE Healthcare). Image analysis was performed using SameSpots software (Totallab, Newcastle, UK). The calculated volume of each spot was normalized against the volume of the Cy2 labeled internal standard spot. In order to investigate changes in the proteome induced by TAM, the spots derived from CPA and CPA + TAM samples were matched. The spots with significant abundance changes (*p* < 0.05 and fold change ≥ 1.5; SameSpots software) between (CPA + TAM)- and CPA-treated samples (differentially expressed protein spots; DEPSs) were designated to mass spectrometry for protein identification.

### Protein digestion and MALDI-TOF/TOF analysis

DIGE gels were re-stained using Coomassie Brilliant Blue G-250 (BioRad, Hercules, CA, USA) and the spots of interest (DEPSs) from 2D separations were dissected from the gels. Proteins in these spots were digested, and mass spectrometry (MS) analysis (MALDI-TOF/TOF) was performed as previously described [68]. Statistical probability of the correct prediction of the identified protein (including peptide mass fingerprint and ion scores) was calculated by MASCOT software. Scores above 70 (*p* < 0.05) were considered significant.

## Supplementary Information

**Additional file 1:** **Supplementary Table 1.** Total body weight±SD (g) of tumor-bearing rats used in the experiment.

**Additional file 2:** **Supplementary Table 2.** Summary of sequence read alignments to the reference genome.

**Additional file 3: Supplementary Table 3.** Differentially expressed genes (DEGs) identified in the ovaries of tumor-bearing rats treated with cyclophosphamide (CPA) plus tamoxifen (CPA+TAM) vs. rats treated with CPA alone.

**Additional file 4**: **Supplementary Table 4.** Functional enrichment analysis of differentially expressed genes (DEGs) identified in the ovaries of rats treated with cyclophosphamide plus tamoxifen (CPA+TAM) vs. rats treated with CPA alone.

**Additional file 5: Supplementary Table 5.** Primers and probes used for real-time PCR.

**Additional file 6: Fig. S1.** Real-time PCR validation of four selected differentially expressed genes (DEGs) which were identified by RNA-Seq in the ovaries of rats treated with cyclophosphamide plus tamoxifen (CPA+TAM) vs. rats treated with CPA alone. The same RNA samples were used for real-time PCR and RNA-Seq (*n*=4 biological replicates). Data were expressed as arbitrary units (AU; mean±SEM). Statistical analysis was performed using t-student test, *P<0.05.

**Additional file 7: Fig. S2.** Representative image (an overlay of Cy3 - green and Cy5 –red channel images) of 2D-DIGE separation of proteins isolated from the ovaries collected from rats treated with cyclophosphamide (CPA) plus tamoxifen vs. rats treated with CPA alone. Circles depict differentially expressed protein spots and shortcuts describe proteins identified by mass spectrometry.

## Data Availability

All relevant data are within the paper, its Supporting Information files, and in the BioProject database under accession number: PRJNA640997.

## Abbreviations

2D-DIGE: Two-dimentional differential in gel electrophoresis
BP: “Biological processes” category of gene ontology database
BSA: Bovine serum albumin
b.w.: Body weight
CC: “Cellular components” category of gene ontology database
CHAPS: 3-[(3-Cholamidopropyl) dimethylammonio]-1-propanesulfonate
CPA: Cyclophosphamide
DEGs: Differentially expressed genes
DEPs: Differentially expressed protein spots
DMBA: 7,12-Dimethylbenz[α]anthracene
DTT: Dithiothreitol
ECM: Extracellular matrix
ER: Estrogen receptor
GnRH: Gonadotropin-releasing hormone
GO: Gene ontology
H&E: Hematoxylin and eosin
HCl: Hydrochloric acid
ip: Intraperitoneal
MALDI-TOF: Matrix assisted laser desorption ionization-time of flight
MNU: N-methyl-N-nitrosourea
MF: “Molecular function” category of gene ontology database
NaCl: Sodium chloride
PCA: Principal component analysis
RNA-Seq: RNA sequencing
RT-PCR: Real-time PCR
SDS-PAGE: Sodium dodecyl-sulfate polyacrylamide gel electrophoresis
STRING: Search tool for the retrieval of interacting genes
TAM: Tamoxifen
TUNEL: Terminal deoxynucleotidyl transferase dUTP nick end labeling
