# Supplementary figures and images for: Tamoxifen decreases ovarian toxicity without compromising cancer treatment in a rat model of mammary cancer
Source: BMC Genomics. 2023 Jun 13;24:325. doi: 10.1186/s12864-023-09423-0 (PMC10265842; doi:10.1186/s12864-023-09423-0)

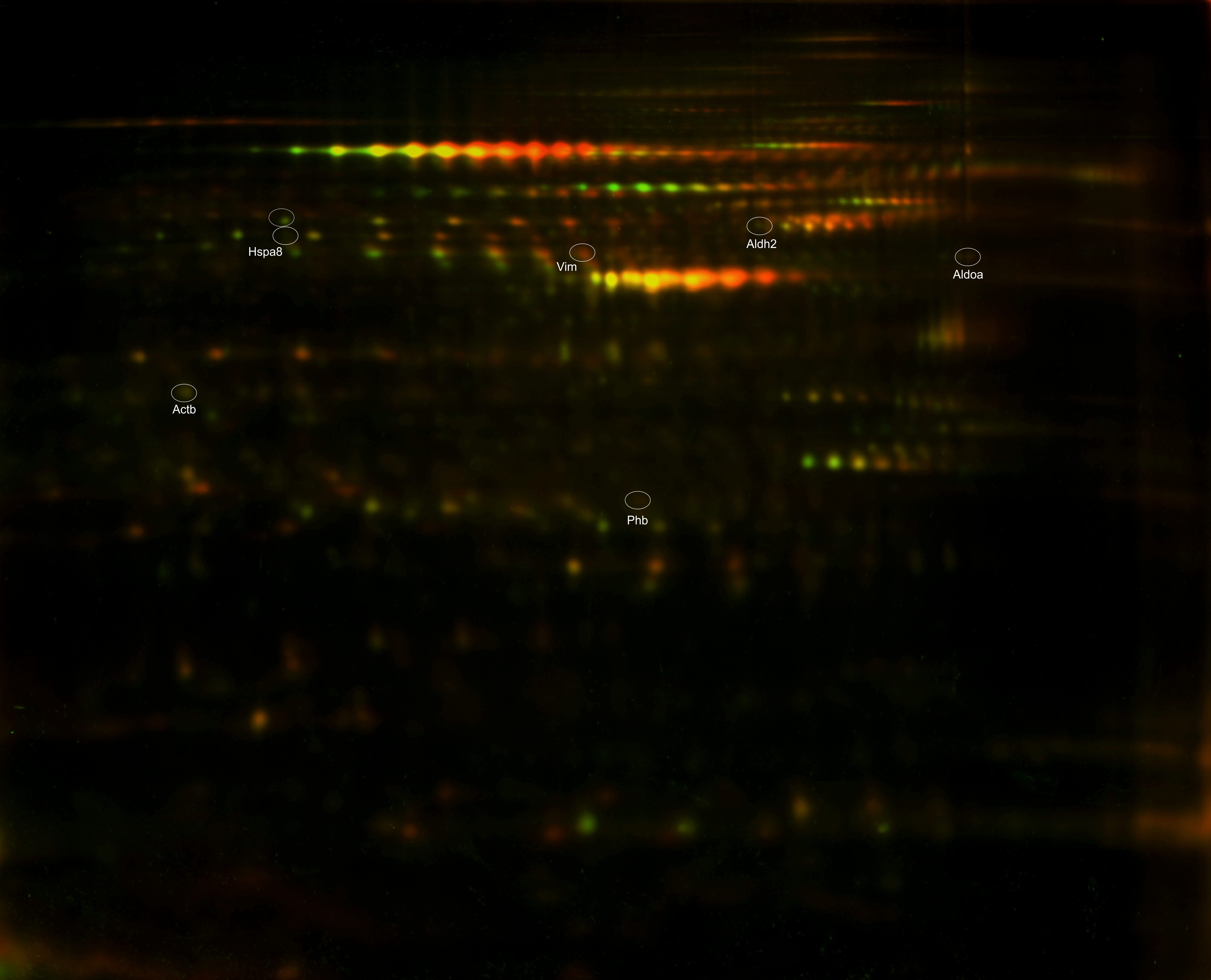

Supplement: Supplementary file 7 — Additional file 7: Fig. S2. Representative image (an overlay of Cy3 - green and Cy5 –red channel images) of 2D-DIGE separation of proteins isolated from the ovaries collected from rats treated with cyclophosphamide (CPA) plus tamoxifen vs. rats treated with CPA alone. Circles depict differentially expressed protein spots and shortcuts describe proteins identified by mass spectrometry. [file 12864_2023_9423_MOESM7_ESM.tif]
